# Role of Serotoninergic Antidepressants in the Development of Autism Spectrum Disorders: A Systematic Review

**DOI:** 10.7759/cureus.28505

**Published:** 2022-08-28

**Authors:** Sheena Mathew, Sumahitha Bichenapally, Vahe Khachatryan, Asmaa Muazzam, Chandani Hamal, Lakshmi Sai Deepak Reddy Velugoti, Godfrey Tabowei, Greeshma N Gaddipati, Maria Mukhtar, Mohammed J Alzubaidee, Raga Sruthi Dwarampudi, Michael Alfonso

**Affiliations:** 1 Pediatrics, California Institute of Behavioral Neurosciences & Psychology, Fairfield, USA; 2 Internal Medicine, California Institute of Behavioral Neurosciences & Psychology, Fairfield, USA; 3 Pathology, California Institute of Behavioral Neurosciences & Psychology, Fairfield, USA; 4 Internal Medicine/Family Medicine, California Institute of Behavioral Neurosciences & Psychology, Fairfield, USA; 5 Research, California Institute of Behavioral Neurosciences & Psychology, Fairfield, USA; 6 Medicine, California Institute of Behavioral Neurosciences & Psychology, Fairfield, USA

**Keywords:** maternal depression, gestational age, pregnancy, selective serotonin reuptake inhibitors, autism specrum disorders, antidepressants

## Abstract

Autism spectrum disorders (ASDs) are one of the most common, highly heritable neurodevelopmental diseases affecting 1-2% of children under the age of 3. Although studies have implicated genetic predispositions, environmental risk factors, and maternal depression as the pathophysiology of ASD, it remains unclear. The association between antidepressant (AD) usage during pregnancy and the likelihood of ASD in children is still debatable. We carried out a systematic review to determine the relation of ASD with AD in offspring exposed to ADs in utero. We used the following terms of medical subject heading (MeSH) and keywords separately and in combination: “antidepressants,” “maternal/pregnancy depression,” “autism spectrum disorders/autism,” and “selective serotonin reuptake inhibitors (SSRI).” Our data search was conducted on PubMed, PubMed Central, Google Scholar, and Cochrane, which resulted in 28,141 articles. We identified and eliminated duplicates and then screened 9,965 articles by title and abstract. We then applied eligibility criteria over 143 relevant articles; a quality assessment was performed, and finally we included 18 selected studies.

Mothers who had taken ADs during pregnancy for at least two medication prescription cycles and children detected to have ASD from two years to 18 years of age were included. We excluded articles in languages other than English, grey literature, case reports, letters to the editor, books, documents, animal studies, and studies published before 2017. Out of 18 studies, 17 evaluated ASD as the primary outcome, and for one study, the outcome was child behavioral as well as neurodevelopmental changes. Other additional outcomes studied were attention deficit hyperactivity disorder (ADHD), preterm birth, spontaneous abortion, small for gestational age, maternal mental illness, and persistent pulmonary hypertension. After adjusting for confounding factors, in six studies, the higher correlations between ASD and ADs were eliminated. Also, paternal AD use, maternal pre-conceptional AD drug use, and maternal depression itself are additional factors that raise the incidence of ASD.

## Introduction and background

Autism spectrum disorder (ASD) is characterized by social impairment, poor communication, stereotypic behavior, aberrant sensitivity to sensory stimuli, and self-injurious conduct [1,2]. The prevalence of ASD has increased over the years from 0.04% to 1% in the United States, and the Centers for Disease Control (CDC) estimates that one in 59 children aged 8 is affected [1,3]. This reasonable increase in the occurrence of ASD can be partially endorsed by better detection and diagnostic criteria, as well as an increase in genetic and environmental factors over time [3,4]. Among several neurodevelopmental disorders, the etiology of autism remains largely unknown and is undoubtedly complex; however, genetic and environmental factors have been linked [4,5].

Pathological findings in ASD include increased oxidative stress, hyperserotonemia, and loss of Purkinje cell integrity in the cerebellum [2]. While the exact cause of autism is unknown, the relation between autism and mental diseases caused by aberrant five-hydroxy serotonin (5-HT) activity, as well as the link between autism and neurological comorbidities caused by 5-HT dysregulation are intriguing [5]. According to the serotonin theory of autism, fetal exposure to high serotonin levels can lead to autism spectrum conditions [6]. High amounts of maternal blood serotonin, for example, could penetrate the developing brain of the baby and cause a loss of serotonin receptors through a negative feedback process, which is also known as the developmental hyperserotonemia (DHS) model of autism. That could explain partly why individuals with ASD have higher blood levels of serotonin as well as decreased activity, synthesis, and binding potential of serotonin in numerous brain locations, according to studies [7].

In some circumstances, identifying modifiable environmental risk factors may benefit primary prevention. The prospect of uncovering one such modifiable causative factor has been raised by some reports suggesting that the usage of selective serotonin reuptake inhibitor (SSRI) antidepressants (ADs) during pregnancy could be related to ASDs in offspring. The importance of the serotonergic system in the pathophysiology of autism is gaining popularity, and prenatal exposure to serotonergic drugs is one biologically plausible avenue [4]. Because the present prevalence of maternal use of a range of drugs has been continuously growing since the 1970s, prenatal exposure to medication may be a factor involved in the risk for ASD [8]. AD use by mothers during the first trimester was less than 1% in 1990, but by 2008, it had risen to over 7%, and nowadays it is 10%, with the majority of ADs being selective serotonin reuptake inhibitors [6,8,9], and mirroring a secular rise in the prevalence of ASD [4].

ADs are used to treat depression and anxiety disorders, which can be influenced by stressful life events [7]. Up to 20% and 15% of women have depression and anxiety during pregnancy, respectively. Pregnant women with untreated depression are at an increased risk for poor obstetric and neonatal outcomes [2]. Given the serious known consequences of these common psychiatric conditions for mothers and their newborns (particularly depression, which is the leading cause of maternal mortality, surpassing hemorrhage, and hypertensive disorders), treatment for prenatal depression appears essential for clinicians [2,10]. Depression treatment combines acute phase management and maintenance phase management; these medications are taken for lengthy periods (at least six to eight months) to achieve treatment goals. ADs used during pregnancy have been shown to cross the placenta, present in cord blood, and create an unfavorable intrauterine environment connected to suboptimal fetal growth, epigenetic changes, and a significant risk of neurodevelopmental disability [7,9].

According to animal research, in utero exposure to serotonergic ADs such as SSRIs may result in faulty serotonin signaling and loss of serotonin terminals in exposed fetuses, as well as changes in brain structure and function, which can lead to negative behavioral effects, culminating in autism phenotype [6,7]. Perinatal SSRI exposure has been shown to reduce the plasticity of the developing hippocampus, particularly in male fetuses, affecting behavioral development and increasing susceptibility to chronic neurodevelopmental disorders later in life [9]. AD medication during pregnancy has been related to ASD in children. Several studies have looked into this theory, but the results have been varied and conflicting [11]. Serotonin-related genetic defects have been found as a possible underlying genetic vulnerability that may be increased by prenatal SSRI exposure, resulting in neurological alterations in the fetal brain associated with an ASD phenotype. However, this is hypothetical, since driving maternal SSRI usage and maternal depression play a complex and potentially confounding function as an independent risk factor for increased ASD symptomatology in offspring [12,13]. A relationship between maternal AD medication and child ASD could be attributable to a direct effect of the drug or external factors between the AD treatment and the outcome [7]. The link between AD usage during pregnancy and the likelihood of ASD in offspring is still debated. Although studies have linked genetic predispositions, environmental risk factors, and maternal depression to ASD, the exact cause is unknown [14].

Some studies also suggest that psychiatric diseases share genetic determinants. Pregnant women who suffer from a mental illness during pregnancy for which ADs may be prescribed have a hereditary vulnerability that children may inherit. As a result, the higher risk of ASD in children of women who used ADs during pregnancy may be explained partially by genetic susceptibility rather than medication. Previous research on the relationship between maternal AD medication and ASD in offspring has been limited to investigations of ADs in general or SSRI ADs, resulting in limited information regarding the risk associated with a non-SSRI group of ADs [4,14].

Even though the majority of studies revealed evidence of correlation, conclusions varied due to concerns about confounding by indication. This was due to the possibility that depression or other psychiatric indications for AD use could be linked to autism via genetic or non-genetic pathways, and thus the possibility that the observed associations represent the risk of autism from the underlying maternal mental illness itself [15]. Reports of linkage between various exposures and the risk of ASD are expected to continue to generate mixed factors as etiology until risk factors for ASD are well understood [14]. As the findings of these association studies are published and added to the body of knowledge, it is critical to not just proceed with caution when considering potential exposure issues but also analyze the findings in light of previous evidence and clinical significance [15]. When it comes to prescribing ADs to depressed pregnant women, having a better awareness is important because it could lead to better outcomes in these vulnerable populations. That is why we conducted our research to inquire about the pros and review cons of maternal AD treatment and its possible relation with ASD in the offspring.

Methods

*Search Strategy* 

**Figure 1:**
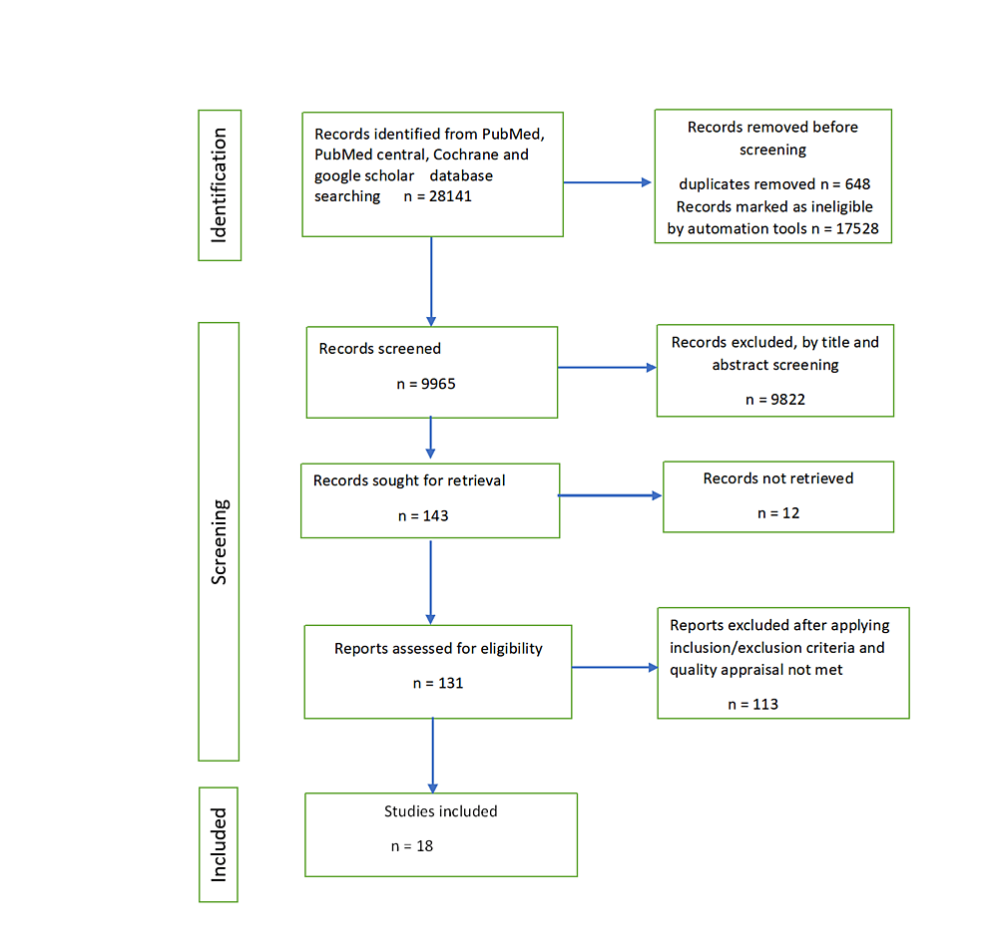
PRISMA diagram showing the study selection process PRISMA: Preferred Reporting Items for Systematic Review and Meta-Analyses

We followed the Preferred Reporting Items for Systematic reviews and Meta-Analyses (PRISMA) guidelines in our systematic review [16]. Articles published in English from 2017 to 2022 were included in this systematic review. Information was obtained with the help of keywords and medical subject heading (MeSH) terms such as “antidepressants,” “selective serotonin reuptake inhibitors,” “gestational/maternal depression,” “autism,” and “development disorder” separately and in combination. Databases used were PubMed, PubMed Central, Cochrane, and Google Scholar. Figure 1 shows the PRISMA flow diagram.

Inclusion Criteria

Mothers who had taken ADs during pregnancy for at least two medication prescription cycles and children detected to have ASDs from 2 years to 18 years of age were included. And studies published from 2017 to 2022 were retrieved.

Exclusion Criteria

We excluded articles in languages other than English, grey literature, case reports, letters to the editor, books, documents, animal studies, and studies published before 2017.

Risk of Bias in Individual Studies

Two authors screened the retrieved articles by titles and abstracts and applied quality assessment tools. Studies with scores ranging from 7 to 8 were included in the study. We used the Newcastle-Ottawa bias scale tool for cohort studies, the assessment of multiple systematic reviews (AMSTAR) tool for systematic reviews, and meta-analysis and the scale for the quality assessment of narrative review articles (SANRA) checklist for reviews.

Results

Study Selection

Our data search on PubMed, PubMed Central, Google Scholar, and Cochrane resulted in 28,141 articles. We identified and eliminated duplicates and then screened 9,965 articles by title and abstract. We then applied eligibility criteria and quality assessment tools to 143 relevant articles; we included 18 in the study. Out of 18 studies, 17 evaluated ASD as the primary outcome, and for one study, the outcome was child behavioral as well as neurodevelopmental changes. Two studies revealed no link between ADs and ASD, whereas eight studies found a positive association, out of which six studies revealed significantly reduced association on adjusting for controlling factors. Paternal AD use, periconceptional AD use, and maternal depression itself are additional factors that raise the incidence of ASD.

## Review

Discussion

Impact of Depression During Pregnancy

According to epidemiologic studies, the prevalence of depression during pregnancy ranges from 8.5% to 11%, with up to 12.7% of women experiencing a major depressive episode. Prescriptions for ADs during pregnancy have increased considerably in the last 10 to 15 years, with estimates ranging from 4 to 16 times. According to research, untreated maternal depression, anxiety, and discontinuing ADs are associated with risks for both the mother and the growing child. This has been related to significant impairments in mother-child attachment and child development, as well as behavioral difficulties, low social engagement, and poor emotional regulation. As a result, physicians need to consider treating both the mother and the infant for prenatal depression [17].

Serotonin-Induced Alterations in the Fetal Brain

**Figure 2:**
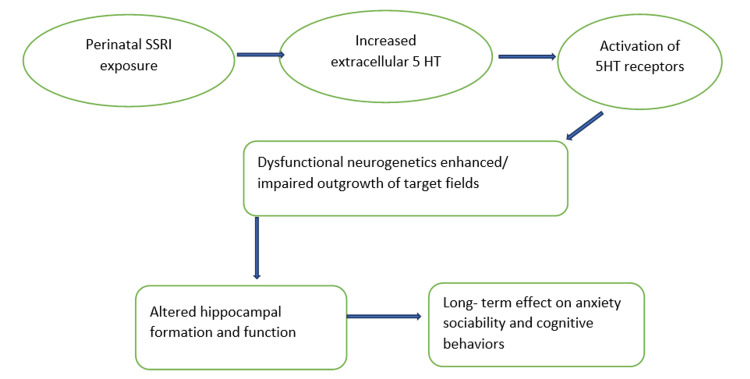
Effects of perinatal SSRI exposure on the fetal brain SSRI, selective serotonin reuptake inhibitor; 5HT, 5-hydroxy tryptamine Figure created by the authors

Because of its efficacy, few side effects, and therapeutic safety, SSRIs are the most prescribed ADs. SSRI medications have been shown to easily pass across the placenta and blood-brain barrier. Neurogenesis, migration, and differentiation, among other elements of brain development, are all influenced by serotonin [17,18]. The indolamine serotonin (5-hydroxytryptamine or 5-HT), in addition to being a neurotransmitter, also serves as a signaling molecule for 5-HT neuron development in the developing brain. Serotonin receptors are visible in human brains by five weeks of pregnancy and increase by 10 weeks [19]. Serotonin levels in the blood are significantly higher in ASD patients. A relation has been demonstrated between prenatal AD drug exposure, abnormal serotonin level in the brain, and motor impairment in infancy and early childhood, and the most evaluated one is ASD [20]. Figure 2, created by the authors, summarizes the effect of serotonin on fetal neurodevelopment.

Studies With Conflicting Results Regarding the Linkage Between AD and ASD

Zhou et al. presented an updated meta-analysis of the relation between maternal AD use during pregnancy and ASD. They included 14 studies, (eight cohort studies and six case-control studies). Pooled adjusted relative risk (RR) for cohort studies (n = 2,839,980) was 1.13 (95% CI: 0.93-1.39), and pooled adjusted odds ratio (OR) for case-control studies (n = 117,737) was 1.51 (95% CI: 1.15-1.99). The main bias was the limited number of studies. However, after adjusting to avoid publication bias, estimates demonstrated a non-significant association (RR for cohort studies: 0.97, 95% CI: 0.79-1.19; OR for case-control studies: 1.26, 95% CI: 0.98-1.62). Hence, the author does not recommend that pregnant woman who has been diagnosed with severe depression stop taking their ADs for fear of having an uncertain risk [21].

From 2002 to 2010, Brown et al. conducted a retrospective cohort analysis of 35,906 singleton births in Ontario, Canada, comparing the risk of autistic spectrum disorder in children exposed to a serotonergic AD in utero to children who were not exposed. The average length of follow-up was 4.95 years, and the average age of the mothers was 26.7 years. The incidence of ASD was 4.51 per 1,000 person-years among children exposed to ADs versus 2.03 per 1,000 person-years among unexposed children (hazard ratio (HR): 2.16 with 95% CI: 1.64-2.86, adjusted HR: 1.59 with 95% CI: 1.17-2.17). The association was not significant (HR: 1.61, 95% CI: 0.997-2.59) after controlling for confounding factors and when compared with unexposed siblings (HR: 1.60, 95% CI: 0.69-3.74). One of the few issues with the study was that it only included women from Ontario's publicly funded lowest socioeconomic groups. Besides, outpatient data were restricted to diagnoses provided by a pediatrician or a psychiatrist to improve precision, and exposure status may have been misclassified if medications were filled but not utilized [13].

A large Swedish cohort study by Rai et al. evaluated 254,610 individuals aged 4-17 years, including 5,378 with autism, living in Stockholm County from 2001 to 2011. Autism was diagnosed in 4.1% (n= 136) of the 3,342 children exposed to ADs during pregnancy compared to 2.9 % (n= 353) of the 12,325 children not exposed to ADs whose mothers had a history of psychiatric disease (adjusted OR: 1.45, 95% CI: 1.13-1.85). The results of propensity score analysis and sibling control analysis were similar; however, only autism without an intellectual disability was linked to an increased risk in all of the studies. As a limitation, it was not possible to investigate trimester-specific or dosage response effects due to the limited sample size and the lack of comprehensive measures of depression severity assessment [22].

ASD, attention deficit hyperactivity disorder (ADHD), preterm birth, persistent pulmonary hypertension, and spontaneous miscarriage were among the outcomes examined by Uguz and he included nine meta-analyses looking at the usage of any form of AD, and ASD was an outcome in 10 of the total studies. In AD exposure groups, the OR for the risk of autistic spectrum disorders was 1.22 to 1.81 for SSRIs, 1.13-1.81 for any AD, and 2.05 for non-SSRIs. Although this evaluation of current meta-analyses suggests that maternal AD usage is associated with a somewhat elevated risk of newborn and childhood outcomes, it is difficult to say if these effects are independent of underlying maternal psychiatric illnesses [23]. Sujan et al. looked at 22 observational studies and found that 10 ASD studies had a pooled adjusted RR of 1.5 (95% CI: 1.3-1.8) for AD exposure. Indeed, evidence from research with a variety of designs and techniques suggests that reported links between prenatal AD exposure and neurodevelopmental difficulties in humans do not provide solid evidence for a causal pathway and could be mostly attributable to confounding factors. The lack of class-specific and trimester-specific studies in addition to the lack of generalizability and presence of confounding factors were limitations of the study [7].

An observational study by Ackerman et al. investigated prenatal AD exposure, maternal depression, the presence of an LGD (likely gene disrupted) mutation, and their interactions with ASD severity. The study included 2,748 patients with ASD, with an average age of 9. In conclusion, researchers discovered that AD exposure and the presence of an LGD mutation had a significant influence on ASD severity. A "two-hit" model is proposed, in which one variable establishes the basis for an initial risk that is then increased by a second variable. However, the small sample size and lack of information regarding out-of-study genetic variations were limitations of the study [12]. In Table 1, we summarize the selected studies describing the varied association between AD and ASD.

**Table 1:** Summary of the selected studies describing the varied association between AD and ASD AD, antidepressant; ASD, autism spectrum disorder; CI, confidence interval; LGD, likely gene mutation; OR, odds ratio; RR, relative risk

Study author and year	Study type, sample size (n)	Outcome and conclusion
Zhou et al., 2018 [21]	Meta-analysis, cohort studies n = 2,839,980, case-control n = 117,737	Cohort studies (RR 0.97, 95% CI: 0.79-1.19) and case-control studies (OR 1.26, 95% CI: 0.98-1.62) demonstrated a non-significant association
Brown et al., 2017 [13]	Retrospective cohort, n = 35,906	The association was not significant for the exposed group (HR: 1.61, 95% CI: 0.997-2.59) and also when compared with unexposed siblings (adjusted HR: 1.60, 95% CI: 0.69-3.74)
Rai et al., 2017 [22]	Cohort, n = 254,610, ASD, n = 5,378	Adjusted OR: 1.45, 95% CI: 1.13-1.85, autism without an intellectual disability was linked to an increased risk
Uguz, 2021 [23]	Review, 9 studies	OR of ASD was 1.13 to 1.81 for any AD, it is difficult to say if these effects are independent of underlying maternal psychiatric illnesses
Sujan et al., 2019 [7]	Review, 22 studies	RR of ASD: 1.5, 95% CI: 1.3-1.8 for AD exposure, also suggests that reported links between prenatal AD exposure and neurodevelopmental difficulties in humans do not provide solid evidence for a causal pathway
Ackerman et al., 2017 [12]	Observational study, ASD, n = 2,748	It postulated that antidepressant exposure and the presence of an LGD mutation have a significant influence on ASD severity

Potential Variables Affecting the Outcome

The European Medicines Agency's (EMA) Pharmacovigilance Risk Assessment Committee (PRAC) concluded in September 2016 that the current evidence does not support a causal relationship and that the available studies on the risk of ASD after in utero SSRI exposure are contradictory, in part due to the different study designs and study populations chosen for analysis. Additionally, Morales et al. performed a systematic review of 15 observational studies (involving 3,585,686 children and 40,585 cases) examining the risk of ASD associated with maternal AD exposure during pregnancy. Pooled effect estimates for the risk of ASD associated with maternal AD exposure during pregnancy compared to unexposed women with a history of affective disorder did not appear to be statistically significantly increased (RR: 1.18, 95% CI: 0.91-1.52), whereas the risk of ASD associated with maternal AD exposure during the pre-pregnancy period compared to all unexposed women appeared statistically significantly elevated (RR: 1.48, 95% CI: 1.29-1.71) [24].

Another systematic review and meta-analysis by Mezzacappa et al. revealed a link between maternal AD usage and the likelihood of autistic spectrum disorder during pregnancy; however, the association appears to be more consistent throughout the preconception period than during each trimester [25]. Six case-control studies (117,737 patients) found a positive link between AD exposure and ASDs during pregnancy (OR: 1.81; 95% CI: 1.49-2.20). When past maternal mental illness was considered, the link was weaker (OR: 1.52, 95% CI: 1.09-2.12). Whatever trimester of exposure was investigated, the same tendency was discovered. There are some disadvantages to this analysis, such as the small number of eligible studies and the inconsistent results of some research [25]. Hagberg et al. performed a cohort study with nested sibling case-control analysis. Among 194,494 mother-baby pairings from UK Clinical Practice Research Datalink (CPRD), they found 2,154 children with ASD. The risk of ASD was 1.72 (95 % CI: 1.54-1.93) for treated depression and 1.50 (95 % CI: 1.28-1.75) for untreated depression, while no increased risk (RR: 0.73, 95% CI: 0.41-1.29) in women who got ADs for other reasons. The results of the sibling analysis were similar to the main analysis. Additional analyses revealed that the risk of ASD in offspring rises with increasing severity, longer duration of depression, and two or more different types of ADs, although, the rates were slightly higher among children of women who were treated for depression [26].

Yamamoto-Sasaki et al. conducted a retrospective cohort analysis to see if AD use was associated with ASD. From January 2005 to July 2014, they collected data on women and children, and children who were followed for 24 months after birth or until their withdrawal from the database. ADs were taken by 195 mothers of the 26,925 eligible mother-child pairs. The mean maternal age at birth of AD users and non-users was 32.1 and 31.4 years, respectively, and 81.5% of AD users were diagnosed with depression during pregnancy. According to a preliminary investigation, the prevalence of ASD is greater with any AD usage than without (OR: 2.32, 95% CI: 1.08-4.95). However, statistical significance was lost when the analysis was controlled for the confounding effect of maternal depression during pregnancy (OR: 0.76, 95% CI: 0.27-2.18). This finding backs up the theory that AD usage during pregnancy does not increase the risk of ASD in offspring. The paucity of socioeconomic data, the small number of women in our sample who used ADs during pregnancy, and the lack of data before the mother's pregnancy are all drawbacks [27].

Another retrospective cohort study on the Swedish offspring (1, 580,629) by Sujan et al. revealed conflicting results. At the population level, first-trimester exposure was associated with an increased outcomes ratio (HR: 2.02, 95% CI: 1.80-2.26) compared with unexposed offspring; however, after accounting for confounding factors, no increased risk of autism was noted in exposed children (HR: 0.83, 95% CI: 0.62- 1.13). The majority of ADs were SSRIs, and the study focused solely on the first trimester, which was considered a limitation of the study [28]. According to a review by Houssari et al. from Ottawa University, there is a possible increase in the risk of ASD in children whose mothers have been exposed to SSRIs. However, the observed results may be confounded by the indication and severity of the mental illness. In this analysis, four studies found higher associations of AD with ASD especially; the use of SSRI during the second and third trimesters was statistically coupled with an elevated risk of ASD. After controlling for confounders, the link between SSRI exposure and ASD was not significant in studies that showed an association. Lack of knowledge about the severity of depression, maternal health, and genetic predisposition to ASD were some of the limitations of the study [29].

Despite the discrepancies in the methodology used by the six meta-analyses, the conclusions were very consistent, according to Andrade. In general, AD use during pregnancy was linked to a higher risk of ASD in children. In studies that accounted for confounding variables and controlled for maternal mental illness; however, the magnitude and significance of the findings were reduced. ADs were also linked to a higher risk even when exposure was limited to the preconception period when the drugs could not have harmed the fetus. One of the most prominent limitations of meta-analyses is that they do not account for multiple hypothesis testing [15]. Table 2 summarizes the studies showing various controlling factors that have been affecting ASD.

**Table 2:** Studies showing various controlling factors that have been affecting ASD AD, antidepressant; ASD, autism spectrum disorders; CI, confidence interval; HR, hazard ratio; MA, meta-analysis, OR, odds ratio; RR, relative risk; SR, systematic review; SSRI, selective serotonin reuptake inhibitor

Study author and year	Type and sample size (n)	Outcome and conclusion
Morales et al., 2018 [24]	SR, 15 studies, n = 3,585,686, ASD, n = 40,585	AD use was not associated with increased risk (RR: 1.18, 95% CI: 0.91-1.52), whereas the AD during the pre-pregnancy period appeared statistically elevated RR: 1.48, 95% CI: 1.29-1.71)
Mezzacappa et al., 2017 [25]	SR and MA, case-control studies, n = 117,737 patients	OR: 1.81, 95 %CI: 1.49-2.20; when past maternal mental illness was considered, the link was weaker OR: 1.52; 95% CI: 1.09-2.12).
Hagberg et al., 2018 [26]	Cohort, n = 194,494, ASD, n = 2154	RR: 1.72, 95 % CI: 1.54-1.93 for treated depression and 1.50,95 % CI: 1.28-1.75 for untreated depression, while no increased risk (RR: 0.73, 95% CI: 0.41-1.29) for those who got ADs for other reasons. The risk was slightly higher among children of women who were treated for depression; however, risk also increases with severity, duration, and the number of ADs.
Yamamoto-Sasaki et al., 2019 [27]	Retrospective cohort, n = 26,925	OR: 2.32, 95% CI: 1.08, 4.95, and when controlled for maternal depression during pregnancy OR: 0.76, CI: 0.27- 2.18, suggesting that AD use alone does not raise the ASD risk
Sujan et al., 2017 [28]	Retrospective cohort, n = 1,580,629	First-trimester exposure HR: 2.02, 95% CI: 1.80- 2.26, after accounting for confounding factors, HR: 0.83, 95% CI: 0.62-1.13
Houssari et al., 2017 [29]	Review	Four studies found higher associations of AD with ASD, whereas, after controlling for confounders, such as the severity of depression, the link between SSRI exposure and ASD was not significant
Andrade., 2017 [15]	Review	AD use was linked to a higher risk of ASD in children, and on accounting for confounding variables such as maternal mental illness, the significance of the findings was reduced. Pre-conceptional AD was also linked to a higher risk.

Is a Group-Specific Study Required for Better Analysis?

Kaplan et al. conducted a meta-analysis of four cohort studies to see if there was a link between prenatal SSRI exposure and ASD in children. The pooled effect size in the maternal psychiatric disorder with no SSRI exposure cohort ASD risk in children was somewhat larger than that of the SSRI exposure during the pregnancy cohort (OR: 1.81 versus 1.61 and 95% CI: 1.44-2.29 versus 1.16-2.25). The low number of studies included in the meta-analysis is the most significant drawback. Nonetheless, the inclusion of cohort studies, which are less prone to bias when measuring exposures during pregnancy, is set as a major strength of this study [30]. Halvorsen et al. performed a systematic review with a meta-analysis of 20 studies. They found a statistically significant association between in utero exposure to SSRIs and mental or behavioral disorders such as ASD (HR: 1.27, 95% CI: 1.10-1.47). The data from the two relevant cohort studies showed a statistically significant association between SSRI exposure and the development of ASD in the first trimester, (OR: 1.59, 95% CI: 1.27-1.98). It is recommended to exercise caution when prescribing drugs to pregnant women because this correlation may not necessarily be a causal relationship due to the residual confounding by indication [31].

Araujo et al. reviewed 20 studies related to ASD, of which one study evaluated the effect of SSRI. The finding that prenatal exposure to SSRIs/serotonin-norepinephrine reuptake inhibitors (SNRIs) does not predict the risk of ASD is very compelling. It is worth noting that the findings of research strongly suggested that maternal depression, whether treated or not during pregnancy, raises the chance of ASD in the offspring [32]. Leshem et al. performed a meta-analysis to investigate the relationship between SSRI, SNRI, ASD, and ADHD and to look for sources of bias. Eighteen studies were included in the meta-analysis. They found an association between SSRIs/SNRIs prenatal use and the risk for ASD (OR: 1.42, 95% CI: 1.23-1.65). Similar findings were obtained in women who were exposed to SSRIs/SNRIs before pregnancy, representing a statistically significant association with ASD (OR: 1.39, 95% CI: 1.24-1.56), although they were not exposed to those medications in utero. The methodology of the studies included in the analysis is prone to recall and exposure bias, which is one of the study's limitations [33].

Another review by Rotem-Kohavi et al. focused on the neurobehavioral outcomes across childhood associated with prenatal SSRI exposure. They stated that maternal depression and autism may be linked through hereditary processes, making SSRI exposure a secondary risk factor underlying the effect of maternal mood (either using genetic inheritance or an environmental influence). These findings also imply that both SSRI usage and maternal depression appear to influence an infant's long-term emotional and behavioral development, both alone and in combination. Inconsistencies in administrative data collecting and clinical classifications of both ASD and maternal depressive mood symptoms hampered this research. The fact that depression is a chronic illness, as well as the number of episodes that occur during the prenatal and postnatal years, adds to the confusion [34]. Table 3 summarizes the studies that evaluated the association between SSRI and ASD as an outcome.

**Table 3:** Studies that evaluated the association between SSRI and ASD as an outcome AD, antidepressants; ASD, autism spectrum disorders; CI, confidence interval; MA, meta-analysis; OR, odds ratio; SNRI, serotonin-norepinephrine reuptake inhibitors; SR, systematic review; SSRI, selective serotonin reuptake inhibitors

Study author and year	Type and characteristics	Outcome and conclusion
Kaplan et al. 2017 [30]	Meta-analysis, 4 studies	The maternal psychiatric disorder with no SSRI exposure and ASD risk in children was larger than that of the SSRI exposure during pregnancy (OR: 1.81 versus 1.61 and 95% CI: 1.44-2.29 versus 1.16-2.25)
Halvorsen et al. 2019 [31]	SR and MA, 20 studies	Mental or behavioral disorders such as autism spectrum disorder and SSRI (HR: 1.27, 95% CI: 1.10-1.47) do not establish a causal relationship
Araujo et al. 2020 [32]	SR, 20 studies	Maternal depression, whether treated or not during pregnancy, increases the chance of ASD; SSRI or SNRI has no role in predicting ASD
Leshem et al. 2021 [33]	SR and MA, 18 studies	Risk for ASD and SSRIs/SNRIs maternal use (OR: 1.42, 95% CI: 1.23-1.65) and risk of AD and periconceptional use (OR: 1.39, 95% CI: 1.24-1.56)
Rotem-Kohavi et al. 2017 [34]	Review	SSRI usage and maternal depression appear to influence an infant's long-term emotional and behavioral development, both alone and in combination

Limitations

Our systematic review has a few limitations. We selected articles published in English and collected data from the last five years; therefore, additional supporting data might have been missed. Lack of the more complete covariate (different groups of ADs, exposure to drugs other than AD, maternal illnesses, and paternal AD use) association studies, along with conflicting results of some studies are other limitations of this review. There is a need for comprehensive studies with a bigger sample size and including more covariates to obtain a detailed analysis.

## Conclusions

Our systematic evaluation validates the relationship between AD exposure in utero and the risk of ASD, but it raises strong reservations regarding causality because there is no substantial risk increase when the SSRI exposure and non-exposure groups are adjusted for covariates. In literature, maternal serotonin is linked to fetal neurodevelopmental changes; hence, children exposed to AD medicines during pregnancy may have a little higher chance of having impaired motor development. In addition, any incremental risk must be weighed against the negative repercussions of inadequately treating depression and related disorders. These factors must be considered when deciding whether to use ADs during pregnancy, considering the specific patient and family circumstances. Additional research is needed, particularly regarding maternal psychiatric disorders, periconceptional AD use, and paternal AD usage.
